# An integrated approach for efficient biomethane production from solid bio-wastes in a compact system

**DOI:** 10.1186/s13068-015-0237-8

**Published:** 2015-04-11

**Authors:** Haoyu Wang, Yu Tao, Margarida Temudo, Margot Schooneveld, Henk Bijl, Nanqi Ren, Monika Wolf, Cornelia Heine, Anne Foerster, Vincent Pelenc, Joris Kloek, Jules B van Lier, Merle de Kreuk

**Affiliations:** State Key Laboratory of Urban Water Resource and Environment, Harbin Institute of Technology, 150090 Harbin, China; Section of Sanitary Engineering, Department of Water Management, Delft University of Technology, 2628 CN Delft, The Netherlands; UNESCO-IHE Institute for Water Education, 2601 DA Delft, The Netherlands; DSM Biotechnology Center, 2600 MA Delft, The Netherlands; Biopract GmbH, 12489 Berlin, Germany

## Abstract

**Background:**

Solid bio-wastes (or organic residues) are worldwide produced in high amount and increasingly considered bioenergy containers rather than waste products. A complete bioprocess from recalcitrant solid wastes to methane (SW2M) via anaerobic digestion (AD) is believed to be a sustainable way to utilize solid bio-wastes. However, the complex and recalcitrance of these organic solids make the hydrolysis process inefficient and thus a rate-limiting step to many AD technologies. Effort has been made to enhance the hydrolysis efficiency, but a comprehensive assessment over a complete flow scheme of SW2M is rare.

**Results:**

In this study, it comes to reality of a complete scheme for SW2M. A novel process to efficiently convert organic residues into methane is proposed, which proved to be more favorable compared to conventional methods. Brewers’ spent grain (BSG) and pig manure (PM) were used to test the feasibility and efficiency. BSG and PM were enzymatically pre-hydrolyzed and solubilized, after which the hydrolysates were anaerobically digested using different bioreactor designs, including expanded granular sludge bed (EGSB), continuously stirred tank reactor (CSTR), and sequencing batch reactor (SBR). High organic loading rates (OLRs), reaching 19 and 21 kgCOD · m^−3^ · day^−1^ were achieved for the EGSBs, fed with BSG and PM, respectively, which were five to seven times higher than those obtained with direct digestion of the raw materials via CSTR or SBR. About 56% and 45% organic proportion of the BSG and PM can be eventually converted to methane.

**Conclusions:**

This study proves that complex organic solids, such as cellulose, hemicellulose, proteins, and lipids can be efficiently hydrolyzed, yielding easy biodegradable/bio-convertible influents for the subsequent anaerobic digestion step. Although the economical advantage might not be clear, the current approach represents an efficient way for industrial-scale treatment of organic residues with a small footprint and fast conversion of AD.

**Electronic supplementary material:**

The online version of this article (doi:10.1186/s13068-015-0237-8) contains supplementary material, which is available to authorized users.

## Background

The growing worldwide energy demands and concomitant fossil fuels constraints have led to the decades’ pursuit of alternative energy from renewable sources. Methane is an energy-rich component that is formed as the end product during the anaerobic decomposition of organic matter, such as domestic slurries and residues coming from food-processing manufactories. Among many different materials that can be used for biogas production, lignocellulose-rich materials, such as plant wastes, and protein-rich materials, such as animal manure, are highly promising due to their high methane potential [1-3].

It is estimated that the world lignocellulosic biomass fixes tenfold the solar energy amount per year compared to the total yearly energy demand of all humans [4]. Therefore, in principle, lignocellulosic biomass could play an increasingly important role in the world future energy production. Brewers’ spent grain (BSG) is largely produced along with the increasing production of beer in recent years. About 15- to 20-kg BSG waste are generated from 1 hL of produced beer, and worldwide, about 1.85 billion hL of beer is produced annually [5]. Animal manure can be considered an even more important energy-containing organic waste, which is also largely produced worldwide. For example, China produces about two billion tons of livestock and poultry manure annually [6]. The number for USA has exceeded one billion tons since 2005 [7]. Anaerobic digestion (AD) of manure can improve the fertilizer value due to enhanced nutrient availability and reduction of the number of pathogens [8]. Moreover, application of manure digestion leads to the recovery of methane as energy source and, when properly applied, reduction of untended emissions. In Europe, AD is regarded the most favorable way for bioenergy production in terms of CO_2_ emission reduction among the various biofuel production possibilities [9]. In addition, considerable amounts of nutrients such as nitrogen, phosphorous, and potassium are mineralized during AD, which can be subsequently reused for agriculture purposes [10].

A bottleneck of applying AD on plant and livestock wastes is the slow rate of hydrolysis because of the complex and recalcitrance of certain components in these materials. Both macroscopic-scale factors, such as tissue compositional heterogeneity and mass transfer limitations, and microscopic-scale factors, such as lignin-carbohydrate cross-linking and cellulose crystallinity, contribute synergistically to the recalcitrance [11]. Direct hydrolysis of this biomass by anaerobic hydrolyzing bacteria is inefficient and regarded as the rate-limiting step in the traditional AD processes [12]. Traditional methanization approaches, such as the use of continuous stirred tank reactor (CSTR), require long residence times in order to meet the slow (rate limiting) hydrolysis step and to prevent the loss of slowly growing microorganisms. A solution is to enhance the hydrolysis step by physicochemical pretreatment, breaking the crystalline structures and promoting access to enzymes for hydrolysis [13-15]. In addition, the solid residues left after pretreatment can be exposed to selective hydrolytic enzyme(s), yielding considerable amounts of protein, glucose, xylose, arabinose, and other compounds from cellulose and hemicellulose, as well as hydrolyzed proteins, into the liquid stream. An integrated approach combining such enzymatic hydrolysis and AD is a promising way for energy recovery from solid substrates. For example, Nkemka et al. performed the digestion of the hydrolysate from pretreated and pre-hydrolyzed wheat straw in an upflow anaerobic sludge blanket (UASB) reactors [16]. Some other previous studies focused more on applying enzymatic hydrolysis to commercial crops for a biorefinery purpose, such as switch grass and corn stover, for bioethanol production [13,17-19]. AD of hydrolysates, for instance, agricultural lignocellulosic wastes, and animal manure is often not considered in these studies.

In this study, two types of biomass, BSG and pig manure (PM), are used to produce methane through a novel approach. In this two-step approach, raw materials are hydrolyzed via a multienzyme pretreatment to convert the chemical oxygen demand (COD)-containing components into a soluble form. After this, the soluble COD is anaerobically converted in high-efficient expanded granular sludge bed (EGSB) reactors to harvest methane. A comprehensive assessment was performed to describe the feasibility, productivity, stability, and energy yield of the mentioned approach by comparing it to CSTRs and sequencing batch reactors (SBRs), which are two traditional anaerobic reactor designs. Our current study includes the research on COD yield after pre-hydrolysis, methane production, volatile fatty acid (VFA) accumulation and utilization, and the impact of salinity and pH.

## Results

### Enzymatic hydrolysis

**Figure 1:**
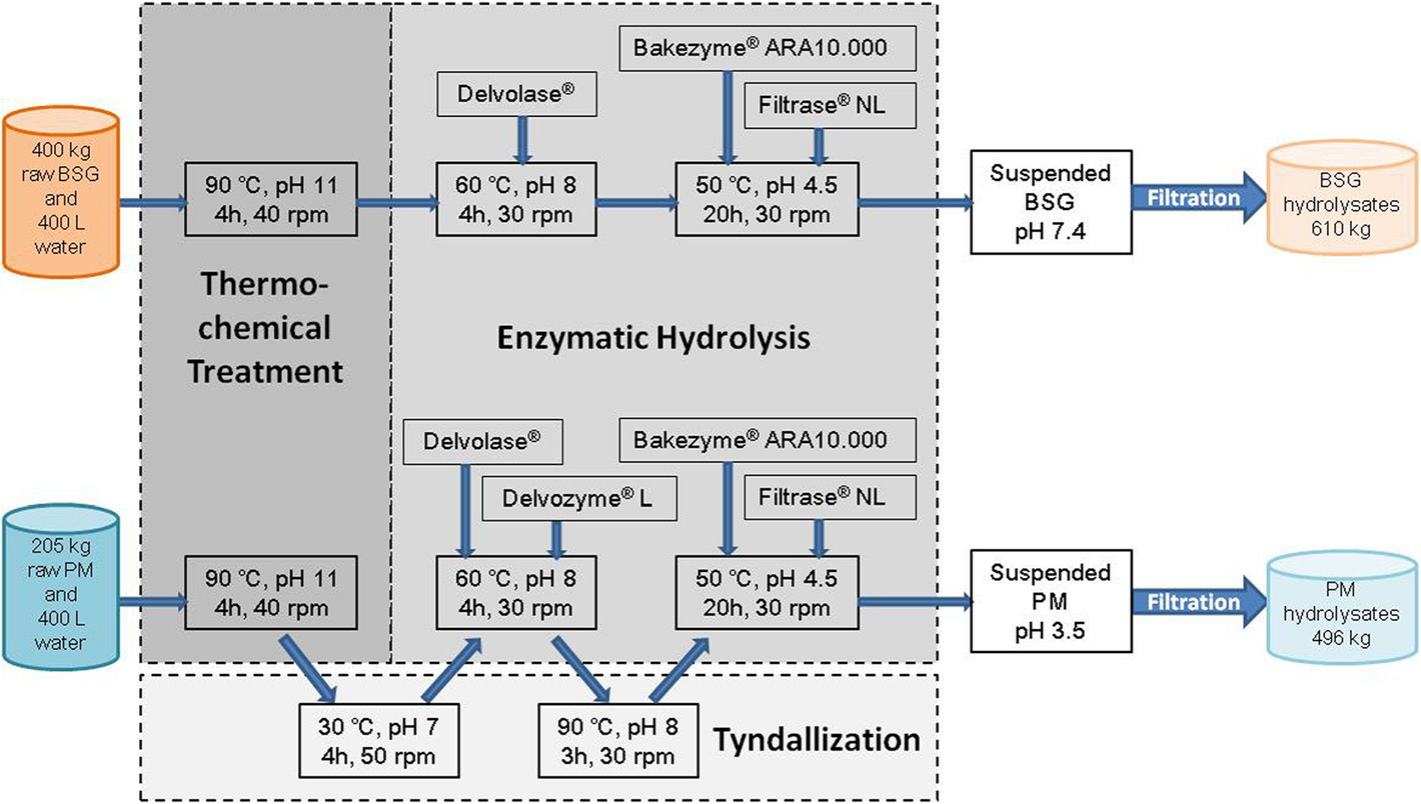
Integrated enzymatic hydrolysis process scheme of raw BSG and PM. Each of Bakezyme®, ARA10.000, and Filtrase® NL was mixed in a total volume of 8 kg solution. BSG, brewers’ spent grain; PM, pig manure.

Multistep enzymatic hydrolysis was applied to hydrolyze raw BSG and PM at a pilot scale (Figure 1). As the raw BSG and PM contain many proteins and lignocellulose, protease, cellulase, and hemicellulase were applied as specific enzymes for hydrolysis. The lysing enzyme was specially used in the PM hydrolysis process to dissolve the cell walls of microorganisms. There were three steps processing the raw BSG and PM: (1) thermochemical pretreatment, which broke down the structure of raw materials to increase the solubilization yield; (2) enzymatic hydrolysis, which was performed by various steps of enzymes under different pHs and temperatures; (3) filtration, which separated the liquid and solid fraction in the last step. The filtered liquid was used as influent (after dilution) for the anaerobic digestion setups and is addressed as hydrolysates. The elemental characterization of raw materials and BSG/PM hydrolysates are shown in Table 1.

**Table 1:** **Characterization of raw BSG, raw PM, and BSG/PM hydrolysates**

**Content (g** · **kg** ^**−1**^ **)**	**Raw BSG**	**Raw PM**	**BSG hydrolysates**	**PM hydrolysates**
Dry matter	199	301	74	58
Organic dry matter	191	233	63	40
Ash	9	78	12	19
Protein	57	48	N.A.	7
Lipids	15	8	N.D.	N.D.
Lignin	29	88	N.D.	N.D.
Total carbohydrates^a^	84	67	15	2
Monomeric glucose	N.D.^b^	N.D.	4.59	0.87
Monomeric xylose	N.D.	N.D.	6.95	0.85
Monomeric arabinose	N.D.	N.D.	3.30	0.56
COD	108	N.A.^c^	100	41
TN	9.15	N.A.	3.54	2.77
Organic nitrogen	N.A.	N.A.	N.A.	1.17
Ammonia-N	0.05	5.00	0.24	1.60
Phosphorus	1.18	N.A.	0.48	0.82
Sulfur	0.59	N.A.	0.27	0.71
Sodium	0.02	N.A.	4.93	3.70
Chloride	0.01	N.A.	2.70	N.A.
Calcium	0.66	N.A.	0.18	2.23
Magnesium	0.36	N.A.	0.12	1.18
Potassium	0.06	N.A.	0.04	1.09

^a^Carbohydrates in raw BSG and raw PM are mainly polysaccharides and so no glucose, xylose, and arabinose monomers were detectable in this study despite of the fact that these sugars were present as building blocks in the polysaccharides. It is clear that all carbohydrates in the hydrolysates of BSG and PM were monosaccharides after degradation. ^b^Not detectable. ^c^Data not available. BSG, brewers’ spent grain; PM, pig manure.

#### Thermochemical pretreatment

**Figure 2:**
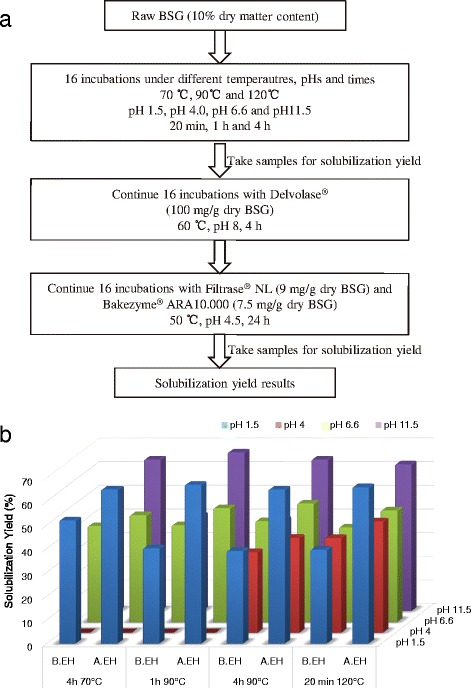
Comparison of solubilization yields under different combinations of temperatures, pHs, and times. (**a**) Scheme of solubilization yields of raw BSG enzymatic hydrolysis process. (**b**) Solubilization yields responding to different conditions. The results of test 4 h 70°C at pH 4 are not available. The solubilization yield was determined using the organic dry matter content of the supernatant and the total slurry after pretreatment (see ‘Calculation of solubilization yield’). *N.A., data not available; A.EH, after enzymatic hydrolysis; B.EH, before enzymatic hydrolysis; BSG, brewers’ spent grain.

The lignocellulosic materials were constructed by the lignin-carbohydrate complexes, in which the biodegradable cellulose and hemicellulose were partially blocked by lignin [20]. Thereof, an appropriate pretreatment is very important to enhance the conversion efficiency from lignocelluloses to saccharides. Temperature and pH are both critical parameters to a successful pretreatment before enzymatic hydrolysis [21,22]. In this study, a series of batch tests was designed (Figure 2a) to compare the effect of different times under different temperatures (4 h 70°C, 1 h 90°C, 4 h 90°C, and 20 min 120°C) and pH conditions (pH 1.5, 4.0, 6.6, and 11.5) on the hydrolysis efficiency of BSG, that is, solubilization yields. Results of solubilization yield (Figure 2b) clearly show that 40% to 50% of the organic dry matter can be solubilized at mild pH conditions (pH 4 and 6.6), whereas 60% to 70% can be solubilized at more extreme pH conditions (pH 1.5 and 11.5). The enzymatic hydrolysis contributes 10% to 20% to the 4-h 70°C pretreatment, while there was 15% to 40% more solubilization derived from enzymatic hydrolysis at 4-h 90°C and 20-min 120°C pretreatments. The extreme pH conditions further benefit enzymatic hydrolysis with a higher improvement on solubilization yields compared to neutral pH conditions. The batch tests proved that the sole thermochemical method (for example, 70°C to 120°C, either pH <2 or pH >11) could contribute to a hydrolysis efficiency in a range of 34% to 52%, while a further enzymatic process can enhance this efficiency to 67%. Considering that an alkaline condition is optimal to the subsequent use of protease, the conditions of pH 11, 4 h, and 90°C were selected for the pilot-scale thermochemical pretreatment (Figure 1).

#### Enzymatic hydrolysis

##### Enzymatic hydrolysis of BSG

Six hundred ten kilograms of liquid BSG hydrolysates was obtained out of a total weight of 800 kg of raw BSG after filtration (Figure 1). The obtained hydrolysates were used to feed the different anaerobic reactor systems, that is, the EGSB, CSTR, and SBR. After the enzymatic hydrolysis, about 70% of the organic compounds from the raw BSG, calculated based on COD, were present in the liquid hydrolysates and 30% was lost in the residual solid. The concentrations of the main components from the raw and liquid hydrolyzed BSG are listed in Table 1. Notably, both lipids and lignin were not detected in the BSG hydrolysates. Results indicate that more recalcitrant matter can be well separated from the hydrolysates, which can greatly benefit the downstream anaerobic digestion process.

##### Enzymatic hydrolysis of PM

In total, 496 kg of liquid PM hydrolysates were obtained from raw PM (Figure 1). The dry matter and organic dry matter concentrations of liquid hydrolysates were only about 47% and 41% of the raw PM (Table 1). More than 91% of carbohydrates and 63% of proteins were converted during the hydrolysis process. The protein concentration decreased by 85%, and lipids and lignin were not detected anymore after the enzymatic hydrolysis. Similar to BSG, the coupling of enzymatic hydrolysis and filtration resulted in liquid section that contains less recalcitrance, likely enhancing the efficiency of anaerobic digestion process.

### Methane yield

#### BMP test

The biological methane potential (BMP) test is used to show the potential methane yield of organic matter, following standardized protocols [23,24]. In our study, the BSG hydrolysates had the highest biogas-production potential value, reaching 810 NmL · gODM^−1^, followed by suspended BSG, which has a biogas-production potential value 680 NmL · gODM^−1^. The raw BSG has the lowest production potential value of 450 NmL · gODM^−1^, which is only 55% of the hydrolysate value. PM hydrolysates also had the highest biogas-production potential value, reaching 485 ± 24 NmL · gODM^−1^ compared to suspended PM and raw PM, which had a biogas-production potential value of 308 ± 10 NmL · gODM^−1^ and 153 ± 8 NmL · gODM^−1^, respectively.

#### Comparison of digestion performance between different reactor configurations

The performance of CSTR, SBR, and EGSB bioreactors was compared with the purpose to determine the most optimal reactor configuration for digesting hydrolysates.

The maximum organic loading rates (OLRs), that is, the ones that could be reached before reactor perturbation, are shown in Table 2. The CSTR results clearly show that pre-hydrolyzed BSG was methanized at twofold higher OLRs compared to the raw BSG. Moreover, results also show that the EGSB reactors fed by hydrolysates were able to run in stable at OLRs as high as 11 kgCOD · m^−3^ · day^−1^ within 3 months after startup and reach to 21 kgCOD · m^−3^ · day^−1^ after 9-month acclimation. The EGSB reactor was characterized by the highest methane production rate, as well as the highest methane yield and the shortest applied hydraulic retention time (HRT). Apparently, the EGSB was the most efficient reactor for digesting BSG hydrolysates compared to CSTR and SBR. In addition to the BSG hydrolysates treatment, the EGSB was also most efficient when treating PM hydrolysates (Table 2). The maximum OLR for the EGSB was in this case seven times higher than the maximum OLR that could be applied to the SBR.

**Table 2:** **Comparison of methane yield in different reactors treating BSG and PM**

**Substrate**	**Reactor type**	**Operational time (days)**	**OLR** ^**a**^ **(kgCOD•m** ^**−3**^ **•day** ^**−1**^ **)**	**OLR** ^**a**^ **(kgODM•m** ^**−3**^ **•day** ^**−1**^ **)**	**Methane yield (L•kgCOD** ^**−1**^ **)**	**Methane yield (L•kgODM** ^**−1**^ **)**	**Methane production rate (mL•L** ^**−1**^ **•day** ^**−1**^ **)**	**HRT (days)**	**Soluble COD removal (%)**
BSG									
Raw BSG	CSTR (5.0 L)	120	4.0	3.0	153	205	617	15.0	N.A.^b^
Suspended BSG^c^	CSTR (5.0 L)	120	5.3	4.0	150	258	802	15.0	N.A.
BSG hydrolysates	CSTR (5.0 L)	120	6.3	4.5	196	295	1,224	10.0	82
BSG hydrolysates	SBR (5.0 L)	80	7.9	5.6	193	292	1,521	12.1	89
BSG hydrolysates	One-stage EGSB (3.8 L)	80	11.5	N.D.^d^	260	N.D.	3,079	2.5	88
BSG hydrolysates	Two-stage EGSB (3.8 L)	120	19.0	N.D.	253	N.D.	4,864	1.5	87
PM									
PM hydrolysates	SBR (5.0 L)	80	3.0	3.3	83	140	249	12.0	91
PM hydrolysates	EGSB (3.8 L)	280	21.0	N.D.	275	N.D.	5,456	1.5	93

^a^The OLR values were the maximum values that were achievable by each reactor, meanwhile the reactors were under stable operational under such OLR conditions. ^b^Data not available. ^c^The mixture of the solid and liquid fraction of hydrolyzed BSG. ^d^Not detectable. BSG, brewers’ spent grain; COD, chemical oxygen demand; EGSB, expanded granular sludge bed; OLR, organic loading rate; PM, pig manure.

#### Methane yield from hydrolysates in EGSBs

**Figure 3:**
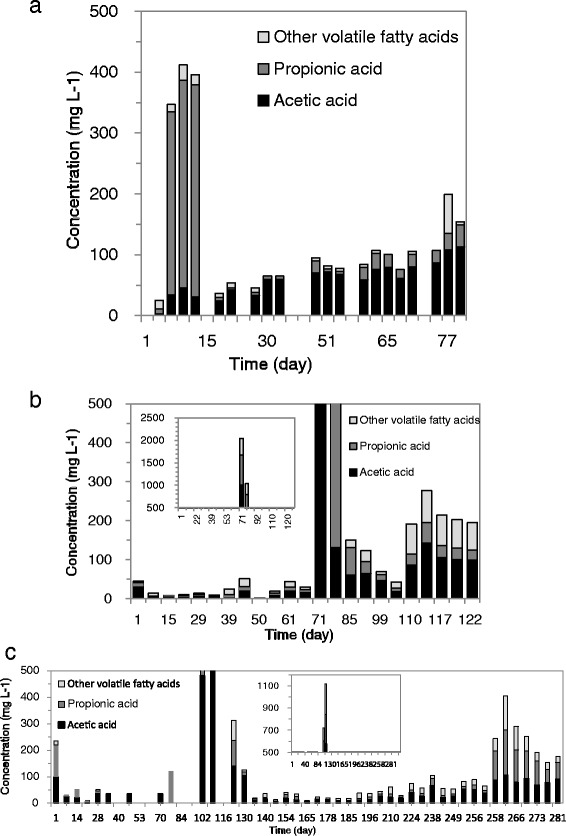
VFA concentrations in the one-stage BSG-EGSB (**a**), two-stage BSG-ESGB (**b**), and PM-EGSB (**c**).

Accumulating VFAs in reactor effluents is generally associated with instability of the AD process [25,26]. A rapid VFA increase might be followed by a subsequent period with low methane production rates [27]. Long-term VFA accumulation and concomitant lack of methane production can even cause a serious drop in pH and may lead to biomass washout and deterioration of the AD process. In our present study, the maximum applicable OLR was searched for, imposing rapid OLR increases to the system. As a consequence, VFA accumulation appeared in all EGSB reactors (Figure 3). The EGSB process stability could be easily recovered in all reactors after temporary OLR decrease, when VFAs was indeed observed in the effluent.

**Figure 4:**
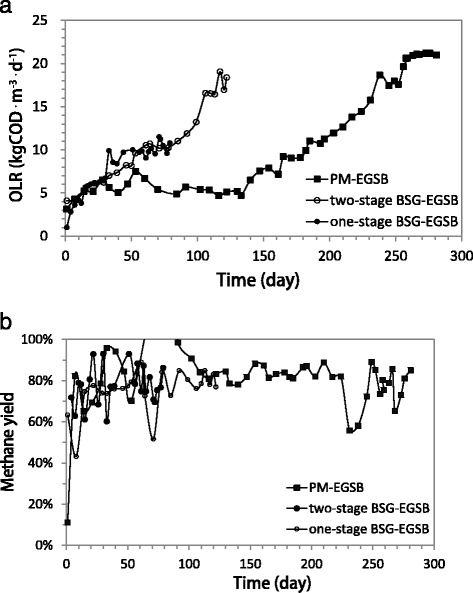
**Organic loading rates (OLRs) and methane yields of the one-stage BSG-EGSB (white circle), two-stage BSG-ESGB (black circle), and PM-EGSB (black square).** BSG, brewers’ spent grain; EGSB, expanded granular sludge bed; PM, pig manure.

The average methane yield, that is, gCH_4_-COD/gCOD_depleted_, for the one-stage BSG-EGSB, two-stage BSG-ESGB, and PM-EGSB was about 79%, 78%, and 81%, respectively. The methane yield of the one-stage BSG-EGSB fluctuated between 60% and 95% in the first month of operation (Figure 4b). A relatively low methane yield, that is, less than 60% was observed in the two-stage BSG-ESGB 1 week after the start-up, which also appeared after the high and sudden OLR increase at days 64 to 71 (Figure 4b). The contribution of the added enzymes to the overall COD of hydrolysates was approximately less than 3.5% and 4% for BSG and PM, respectively, while the released nitrogen only accounted for about 1.2% and less than 1% of the total nitrogen of the BSG and PM hydrolysates, respectively.

The seeding sludge for the three EGSBs was characterized by a high specific methanogenic activity (SMA) of 0.73 to 1.2 gCOD_CH4_ · gVSS^−1^ · day^−1^. Nonetheless, reactors were started at low OLRs of 0.1 to 0.2 gCOD · gVSS^−1^ · day^−1^, in order to facilitate the adaptation of microbial communities. The applied OLRs at the start of the continuous flow experiment were less than a quarter of the biogas producing capacity of the inoculum. Subsequently, OLRs were increased stepwise, according to the performance of each EGSB. The monitored indicators that were used for assessing the reactor stability were methane COD conversion efficiency and VFA accumulation. The inoculums could easily adapt to BSG hydrolysates, resulting in a more rapid increase in OLR applied to the BSG-EGSBs compared to the PM-EGSB (Figure 4a).

Large amount of aceticlastic methanogens were expected in the seeding sludge as it showed very high values in the SMA tests, in which acetate was used as the sole carbon source. In order to understand if the applied sludge loading rates (expressed as gCOD · gVSS^−1^ · day^−1^ of biodegradable hydrolysates) were appropriate, SMA tests were applied to both BSG-EGSB and PM-EGSB sludge at different periods, more (Table 3). The results showed that the applied sludge loading rates were higher than the corresponding SMAs of the PM reactor sludge, but were close to the corresponding SMAs of the BSG reactor sludge, except for the starting period of the two-stage BSG-EGSB (Table 3). Interestingly, the observed seemingly *overloading* of PM biodegradable hydrolysates did not lead to unstable methane production or reactor perturbation. It is very likely because the sludge SMA values were assessed with acetate as the sole substrate, which missed the potential contribution of hydrogenotrophic methanogens. As a matter of fact, by combining the results of 454-pyrosequencing and real-time quantitative polymerase chain reaction (see Additional file 1: Supplementary Material), we found in the PM-EGSB that the quantity of hydrogenotrophic methanogens was sometimes two orders of magnitude higher than the amount of aceticlastic methanogens; accordingly, the relative abundance of hydrogenotrophic methanogens was also much higher than that of aceticlastic methanogens (Additional file 1: Table S1), while such differences were small or reversed in both one-stage and two-stage BSG-EGSBs. These results indicate that hydrogenotrophic methanogens very likely played a more important role in the PM reactor compared to the BSG ones. However, such metabolic route cannot be revealed by a standard SMA test because syntrophic associations between acetogens and hydrogenotrophic methanogens are not maximized when acetate is used as the sole carbon source in such tests. These results reminded us of the possible population shift after a period feeding of substrates such as PM hydrolysates, and such change in microbial community may lead to biased (or confusing) results from an unchanged analytical method.

**Table 3:** **Specific methane activity (SMA) of the EGSBs fed with BSG hydrolysates or PM hydrolysates during different periods**

**Date**	**SMA** ^**a**^ **(gCOD**_**CH4**_ **· gVSS** ^**−1**^ **· day** ^**−1**^ **)**	**Biomass-based OLR (gCOD**_**hydrolysates**_ **· gVSS** ^**−1**^ **· day** ^**−1**^ **)**	**Biomass-based OLR (g biodegradable COD**_**hydrolysates**_ **· gVSS** ^**−1**^ **· day** ^**−1**^ **)**	**Overloading rate**
One-stage BSG-EGSB				
Inoculum	0.73 ± 0.08	N.A.^b^	N.A.	N.A.
Day 79	0.49 ± 0.01	0.67	0.48	Not overloading
Two-stage BSG-EGSB				
Inoculum	0.73 ± 0.08	N.A.	N.A.	N.A.
Day 8	0.44 ± 0.01	0.20	0.14	Not overloading
Day 122	0.39 ± 0.02	0.63	0.45	15%
PM-EGSB				
Inoculum	0.73 ± 0.08	N.A.	N.A.	N.A.
Day 8	0.20 ± 0.06	0.47	0.43	115%
Day 105	0.22 ± 0.01	0.33	0.30	36%
Day 140	0.27 ± 0.03	0.42	0.39	44%
Day 263	0.32 ± 0.02	0.76	0.70	119%

^**a**^Measured with acetate as the substrate. ^b^Data not available. BSG, brewers’ spent grain; COD, chemical oxygen demand; EGSB, expanded granular sludge bed; OLR, organic loading rate; PM, pig manure.

VFA accumulation was observed in the one-stage BSG-EGSB immediately following the start-up (Figure 3a). However, there was no such VFA accumulation after the start-up of the two-stage BSG-ESGB. The more stable process performance might be attributed to a higher Archaea/bacteria ratio in the second (EGSB) stage of the two-stage process compared to a single-stage process. However, severe VFA accumulation was observed in the two-stage BSG-ESGB reactor with maximum total VFA concentrations reaching about 2.0 g · L^−1^ during the week of day 70 (Figure 3b), corresponding to a simultaneous drop in methane yield (Figure 4b). The PM-EGSB also experienced severe VFA accumulation with total VFA concentrations exceeding 1.0 g · L^−1^ between days 100 to 120 (Figure 3c). Slightly accumulating VFAs were observed in the PM-EGSB during days 218 to 237 (Figure 3c) followed by a VFA accumulation up to 0.4 g · L^−1^ as total VFA, from day 258 onwards.

## Discussion

High-rate biomethanation of organic residues can be achieved by coupling a separate, enzymatic pre-hydrolysis step to a high-rate anaerobic reactor system. BSG and PM were selected to test the feasibility, productivity, stability, and energy yield of the current approach. Firstly, BSG and PM were treated by enzymatic hydrolysis, whereafter the solubilized hydrolysates was processed in anaerobic CSTRs, SBRs, and EGSBs for biogas production.

One of the critical parameters before/during enzymatic pretreatment is pH. Our results show that the extreme pH conditions of pH <2 or pH >11 could further enhance the enzymatic pre-hydrolysis, measured as solubilization yield. Results also showed that the solubilization yields at neutral pH conditions are quite limited. Our observations are in line with other studies reporting that extreme pH conditions contribute to yield considerable percentages of sugars from hemicelluloses and cellulose and favored the subsequent enzymatic hydrolysis [13,28,29]. Compared to acid pretreatments, a high pH condition could be more preferable because it requires lower temperatures [29] and is more efficient in removing lignin-like materials [30].

Solid waste streams are generally digested using large CSTR type of digester systems that are usually operated with hydrolysis as the rate-limiting step. Solubilizing these solid waste streams creates the possibility to use high-rate wastewater treatment reactors to digest the hydrolysates. In fact, all types of bioreactors can be used. Of the various high-rate systems, EGSB reactors are characterized by a very compact configuration, a small footprint, and an advanced gas/liquid/solid separation device. Moreover, an EGSB reactor is well accepted for industrial applications and, therefore, can be easily scaled up for methane recovery from BSG and PM hydrolysates. In our present research, the solubilized BSG hydrolysates showed a four- to fivefold higher biogas production efficiency in a two-stage EGSB system, compared to the direct treatment of suspended BSG hydrolysates and raw BSG solid waste.

A high treatment efficiency and a high methane yield on hydrolyzed BSG/PM are the two main observations of our current study. Firstly, the EGSBs were characterized by a stable treatment performance applying extreme OLRs, as high as 19 kgCOD · m^−3^ · day^−1^ for pre-acidified BSG hydrolysates and 21 kgCOD · m^−3^ · day^−1^ for PM hydrolysates. The applied OLR could be rapidly increased to 11.5 kgCOD · m^−3^ · day^−1^ within 3 months for the one-stage BSG-EGSB, with a high and stable methane yield. Secondly, the methane yield of the EGSBs is about 80%, meaning that 80% of organic matters in BSG and PM hydrolysates are eventually converted to methane. Considering the high organic yield of the pre-hydrolysis process, that is, about 70% for BSG and 45% for PM, the overall methane yield from raw BSG and PM, including losses during pretreatment, were 56% and 36%, respectively. The enzymatic pretreatment saves considerable time for hydrolysis compared to direct biological hydrolysis by anaerobic bacteria; the solubilized hydrolysates are easier for acidogens to utilize, which will result in more compact anaerobic reactor systems with higher loading potentials than when applying direct digestion. Meanwhile, the current method can maximize the organic conversion efficiency, especially when pretreatment is further improved, minimizing COD losses.

In this study, ordinary anaerobic granules from a food-processing factory were used to inoculate all reactors. It is notable that the CSTR might perform better on digesting the raw BSG or PM if it was inoculated with some types of *more appropriate* seeding sludge. However, the success of our inoculation was a more representative confirmation to the proposed method than inoculating specialized sludge.

A potential downside of the used pH control conditions during pre-hydrolysis is the introduction of considerable amounts of Cl^−^ and Na^+^ into the liquid hydrolysates. The hydrolysate characterization (Table 1) shows that the contents of sodium and chloride in the BSG hydrolysates liquor are 4.93 and 2.70 g · kg^−1^, respectively, which are much higher than their concentrations in raw BSG solids (0.02 and 0.01 g · kg^−1^, respectively). Also in PM hydrolysates, the measured salinity was very high, that is, 13 g · L^−1^, which is distinctly higher than generally found in anaerobic digestion reactors. High salinity, for example, exceeding 10 g · L^−1^, may negatively impact the anaerobic digestion process [31,32] and may lead to weak sludge granules [33,34]. The strength and size of these granules is essential for operating a high-efficiency anaerobic reactor system such as UASB and EGSB [35]. Considering these negative potentials, alternative acids and bases should be tested to overcome salinity-derived problems and even favor the subsequent anaerobic digestion process.

**Figure 5:**
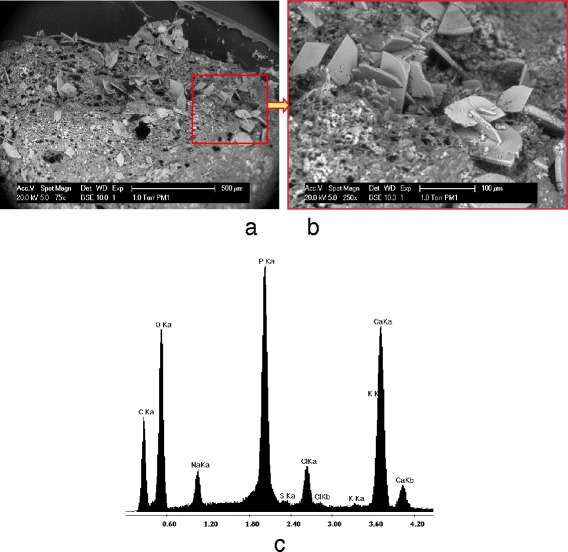
Scanning electron microscopic photo of a PM-EGSB granule (**a**, **b**) and energy-dispersive X-ray analysis (**c**) on the precipitates on the granule.

Another negative issue is the high mineral content in pig manure. It is notable that the calcium and magnesium concentrations in PM hydrolysates are 2.23 and 1.18 g · kg^−1^, which are 12.5 and 9.5 times higher than that in BSG hydrolysates. Bivalent cations can potentially precipitate inside reactors during anaerobic digestion, depending on operational temperatures and pH conditions. In our study, we observed such precipitates on the surface of PM-EGSB granules (Figure 5a) and the energy-dispersive X-ray (EDX) analysis proved that the major mineral elements of these precipitates were sodium and calcium (Figure 5b). The accumulation of precipitates inside an EGSB reactor may cause high total suspended solid (TSS) concentrations and low ratios of volatile suspended solids (VSS) to TSS.

Although this study proves that the concept of high-efficiency methanization of BSG and PM is technically feasible using this integrated approach, the enzymatic hydrolysis processes might not have apparent advantage from the economical perspective. An in-depth economical analysis on the selection of enzymes and operational optimization is necessary, and some alternatives in making this process more economically attractive are needed.

## Conclusions

This paper presents a novel way to methanize two common organic residues, namely BSG and PM, at high efficiency. Firstly, the enzymatic pretreatment helps to break down the rigid solid matrix and to convert the large-molecule organic matter, such as cellulose, hemicellulose, and protein, into small monomers, and the non-hydrolyzed residue, such as lignin, is separated from the hydrolysates by solid–liquid separation. The organic-rich hydrolysate liquor could be transformed into methane via anaerobic digestion in CSTR, SBR, or EGSB reactors. The whole process that is catalyzed by enzymes is shown to have a high yield and a high efficiency. In this proof of concept study, about 56% and 45% of the total organic matter from BSG and PM were eventually converted to methane. Solubilized hydrolysate-fed EGSBs performed stable at OLRs of 19 kgCOD · m^−3^ · day^−1^ (BSG) and 21 kgCOD · m^−3^ · day^−1^ (PM), which is five to seven times higher than conventional reactor systems and methods, applying CSTRs or SBRs directly fed with raw organic solids. Our study demonstrates that the separation of the enzymatic hydrolysis step and the methanation step provides an optimal control and selection of conditions to efficiently treat the organic solids. The proposed technology represents a promising technique for the industrial-scale treatment of organic solids with a high energy yield and a high efficiency but using only a small footprint of AD.

## Materials and methods

### Raw BSG and PM

Raw wet BSG was obtained from a brewery plant in The Netherlands. Raw BSG is a wet slurry, mainly consisting of organic dry matter, consisting of protein, lipids, lignin, and carbohydrates, and an inert ash fraction (Table 1). PM was obtained from a manure trader in The Netherlands. The compositions of raw PM and BSG are listed in Table 1. The main differences between the hydrolysates are the elevated ammonia nitrogen concentration in raw PM (5 gNH_4_^+^-N · kg^−1^), as well as the elevated salinity in PM.

### Enzymatic hydrolysis

#### Enzymatic hydrolysis setup

The enzymatic hydrolysis of BSG and PM was carried out in a stainless steel tank with a working volume of 1,500 L. The reactor was equipped with a cooling/heating jacket, to control the temperature of the slurry (30°C to 95°C). The slurry was stirred with a variable speed anchor type mixer.

#### Enzymes

The enzymes that were used for the hydrolysis of raw PM and BSG were provided by DSM (Delft, The Netherlands). Delvolase® (DSM, Delft, The Netherlands) is an alkaline protease derived from bacteria *Bacillus licheniformis*. The optimum conditions of Delvolase® are pH 8 to 10 at 55°C to 60°C. Delvozyme® L is a purified enzyme extracted from egg white and is able to lyse cell walls of vegetated bacteria, which shares the same optimum conditions with Delvolase®. Filtrase® NL (DSM, Delft, The Netherlands) is a liquid fungal beta-glucanase, cellulase, and xylanase from *Talaromyces emersonii*. Bakezyme® ARA10.000 (DSM, Delft, The Netherlands) is a type of hemicellulase commonly used in bakery process. The optimum conditions for Filtrase® NL and Bakezyme® ARA10.000 match well at pH 4.5 around 50°C.

#### Enzymatic hydrolysis of BSG

The hydrolysis process of BSG is shown in Figure 1a. Firstly, a thermochemical pretreatment was applied to the raw BSG slurry to improve the solubilization yield of organic matters under the conditions of pH 10.7, 90°C for 4 h. And then several steps of enzymatic hydrolysis were applied under the optimum conditions of each enzyme. The pH was adjusted by adding NaOH/HCl. Delvolase® was applied in the first place, which can hydrolyze both native and denatured proteins after the thermochemical pretreatment. After 4 h, Filtrase® NL and Bakezyme® ARA10.000 were added together to break intricate 3D networks of cellulose and hemicellulose. Since the optimum pH of protease (Delvolase®) is higher, it was added prior to cellulase/hemicellulase to avoid too many pH adjustments. Twenty hours later, the pH of slurry was neutralized and then the slurry was filtrated by a multifilament cloth (Sefar Tetex, Bern, Switzerland) to get the liquid fraction for the subsequent AD reactors.

#### Enzymatic hydrolysis of PM

The raw PM contained many microorganisms (such as pathogens and spores), which could overgrow, utilizing the hydrolyzed carbohydrates and decreasing the biomethane potential of the hydrolysates. Hence, a tyndallization was applied to inactivate the microorganisms in PM (Figure 1b). Most cells were inactivated after the thermochemical treatment but spores can still survive. An incubation at pH 7 and 30°C for 4 h was applied to germinate the live spores, and another heat treatment was applied at 90°C for 3 h. Additionally, the pH of PM was adjusted to 3.5 at the end to prevent the growth of microorganisms. The total amount of active cells decreased from more than 1 × 10^8^ CFU/mL in the raw slurry to less than 120 CFU/mL in the PM hydrolysates. The left hydrolysis process of PM was similar to BSG’s process.

### Anaerobic digestion setups

#### Continuously stirred tank reactors

Three identical CSTR reactors (CSTR1, CSTR2, CSTR3) had a working volume of 5 L. They were fed with raw BSG (CSTR1), suspended BSG (CSTR2), and solubilized BSG (CSTR3) hydrolysates, respectively. The operation temperature was 35 ± 1°C. The pH of all CSTRs was controlled in a range of 7.1 to 7.5. The HRT of CSTR1 and CSTR2 was set to 15 days and CSTR3 had an HRT of 10 days.

#### Sequencing batch reactor

Two identical SBRs had a working volume of 5 L. Solubilized BSG and PM hydrolysates were fed to each SBR. The operation temperature was 35 ± 1°C and the HRT was set to 12 days. The pH of the two SBRs was controlled between 7.2 and 7.7.

#### One-stage EGSB

Three identical glass-made EGSB reactors (manufactured by Louwers, Enschede, The Netherlands) with a working volume of 3.8 L were used in this study. Two EGSBs were fed with BSG hydrolysates (BSG-EGSB) and another one was fed with PM hydrolysates (PM-EGSB). The operational temperature was maintained at 35 ± 1°C by a water bath (Tamson Instruments, Bleiswijk, The Netherlands). Peristaltic pumps (Watson Marlow, Cornwall, United Kingdom) were used to feed the reactor with a constant influent flow rate to maintain the HRT to about 1.5 days. An effluent recirculation was used to supply a constant upflow velocity of 8 m · h^−1^. The biogas was collected via a three-phase separator from top of the EGSB, and the biogas flow was constantly measured by a milligas counter (type MGC-1 PMMA, Ritter, Schwabmünchen, Germany). Temperature, oxidation-reduction potential (ORP), and pH were online monitored by sensors (Mettler Toledo, Greifensee, Switzerland), and the pH was controlled to be always above 6.9 by adding 0.1 mM NaOH. LabView software was used to control the pumps and data collection.

#### Two-stage EGSB

A 1.8-L (working volume) pre-acidification bottle was set prior to one BSG-EGSB and the combined system was named two-stage BSG-EGSB. The sludge that was washed out from the one-stage BSG-EGSB was inoculated to the pre-acidification bottle. The HRT was controlled as 8 h by a peristaltic pump and the bottle was operated under ambient temperature (18°C to 24°C). A magnetic stirrer was used to mix the bulk liquid, and some biomass was inevitably pumped into EGSB along with the pre-acidified hydrolysates. The online monitor system for temperature, pH, and ORP was the same as the ones equipped for EGSBs.

#### Inoculum

Anaerobic granules from a full-scale upflow anaerobic sludge bed that treated potato processing wastewater (Germany) were inoculated to all the lab-scale anaerobic digestion reactors (CSTRs, SBRs, and EGSBs) and were used for the BMP tests.

### Analytical methods

#### Basic analysis

TSS, total solids (TS), VSS, and volatile solids (VS) were measured twice per week according to standard methods [36]. COD, ammonia nitrogen and total nitrogen (TN) were measured by the corresponding testing kits (product numbers 1145410001, 114559, and 114763, Merck, Darmstadt, Germany) and spectrophotometer (Spectroquat TR420/NOVA60, MERCK, Merck, Darmstadt, Germany). Each analysis was performed in duplicate. The samples for soluble COD measurement were filtered through a 0.45-μm fiberglass filter (Spartan 30, Whatman, GE Healthcare, Buckinghamshire, United Kingdom) before analysis. The salinity was tested using a conductivity meter. VFAs (C2-acetic acid, C3-propionic acid, C4-butyric acid, iC4-isobutyric acid, C5-valeric acid, and iC5-isovaleric acid) were quantified by gas chromatography (GC, HP7890 Agilent Technologies, Palo Alto, CA, USA) equipped with a flame ionization detector. The GC was fitted with a capillary column (19095 N-123 HP INNOWX). The temperature of column, the injector port, and the detector was 70°C, 250°C, and 300°C, respectively. The carrier gas was nitrogen at a flow rate of 10 mL · min^−1^ and a split flow of 40 mL · min^−1^. The methane content in biogas was measured using a 7890A gas chromatograph (Agilent Technology, USA) with a thermal conductivity detector and a 45- to 60-mesh matrix molecular sieve 5A column (Sigma-Aldrich, St. Louis, MO, USA). Helium gas was the carrier gas at a flow rate of 30 mL · min^−1^. The temperature of the injection inlet, oven, and detector was 100°C, 60°C, and 105°C, respectively.

#### Calculation of solubilization yield

The solubilization yield was determined using the organic dry matter content of the supernatant and the total slurry after pretreatment, using the following equation:

$$ \mathrm{Solubilization}\kern0.5em \mathrm{Yield}\kern0.5em \%\kern0.5em =\kern0.5em \left({\mathrm{ODM}}_{\mathrm{S}}/{\mathrm{ODM}}_{\mathrm{T}}\right)\kern0.5em \times \kern0.5em \mathrm{F}\kern0.5em \times \kern0.5em 100\% $$

in which ODM_S_ is the organic dry matter content of the supernatant (%), ODM_T_ is the organic dry matter content of the total slurry (%), *F* is the correction factor for the pellet volume, which is calculated as:

$$ \mathrm{F}\kern0.5em =\kern0.5em \left(1-{\mathrm{ODM}}_{\mathrm{T}}\right)/\left(1-{\mathrm{ODM}}_{\mathrm{S}}\right) $$

#### Characterization of liquid hydrolysates solution and EGSB effluent

Protein characterization was a combination of precipitation of proteins using trichloroacetic acid (TCA) to remove disturbing substances and allow determination of the protein concentration with the colorimetric Biuret reaction. The standardization was performed using bovine serum albumin (BSA). Carbohydrates and lignin content were determined as described in Sluiter et al. [37].

#### Sludge characterization

##### Specific methanogenic activity

SMA was used to estimate the capacity of methanogenic microorganisms to convert acetate into CH_4_ in the anaerobic system. In this study, the SMA of the EGSB sludge was determined using an Automated Methane Potential Test System (AMPTS, Bioprocess Control, Lund, Sweden). The synthetic media for the control group consisted of a mixture of macronutrients, trace elements, and phosphate buffer solution. This medium was also used for the SMA test itself, supplemented with sodium acetate (2 g · L^−1^) as substrate. The inoculum amount was determined by setting an inoculum VSS to substrate COD ratio (I/S) of 2:1. Both the reactor sludge and control group were analyzed in triplicate. The interpretation of SMA was expressed as kgCOD_CH4_ per kgVSS_sludge_ per day, in order to compare with other studies.

##### BMP test

The BMP was determined using 15-mL hydrolysates added to a 50-mL batch digestion vial. In two independent experiments, 4 replicates of 15-mL hydrolysates were incubated during 21 days at 39°C. Increasing gas pressure in the head space during digestion was measured once per day in the first week, each second day in the second week, and each third day in the last week. Normalized gas production in consideration of methane content that was measured by GC was calculated and compared to corresponding controls, which were the incubations without hydrolysates.

##### ESEM and EDX element analyzing system

ESEM and EDX analysis were applied to observe the surface of PM-EGSB granules and analyze the elemental constitution of precipitates on such granules. The granule samples were freshly sampled and fixed by 2% (*v*/*v*) glutaraldehyde for 2 h before ESEM observation. For ESEM observation, the samples were mounted on a 1-cm^2^ metal support and kept in place with adhesive tape and observed with a Philips XL30 Series ESEM (Philips, Amsterdam, The Netherlands). There was no need to apply a gold sputter coating on the sample, since by using the ESEM, there is a reduced build-up of static electricity. The EDMA-3 system (SUTW 3.3 EDX window and 128.0-eV EDX resolution) was applied to analyze the key elements of precipitates.

## Additional file

Additional file 1: **Supplementary materials.**

## Abbreviations

AD: anaerobic digestion
BMP: biological methane potential
BSG: brewers’ spent grain
CFU: colony forming units
COD: chemical oxygen demand
CSTR: continuously stirred tank reactor
EDX: energy-dispersive X-ray
EGSB: expanded granular sludge bed
ESEM: environmental scan electron microscopy
GC: gas chromatograph
HRT: hydraulic retention time
ODM: organic dry matter
OLR: organic loading rate
ORP: oxidation-reduction potential
PM: pig manure
SBR: sequencing batch reactor
SMA: specific methanogenic activity
TN: total nitrogen
TS: total solids
TSS: total suspended solids
VFA: volatile fatty acid
VS: volatile solids
VSS: volatile suspended solids
