# Supplementary material for: An integrated approach for efficient biomethane production from solid bio-wastes in a compact system
Source: Biotechnol Biofuels. 2015 Apr 11;8:62. doi: 10.1186/s13068-015-0237-8 (PMC4394555; doi:10.1186/s13068-015-0237-8)
Supplement: Supplementary file 1 — Supplementary materials. [file 13068_2015_237_MOESM1_ESM.pdf]

Supplementary Materials

A novel approach for efficient biomethane production from solid bio-wastes in a compact system

Supplementary Text

Sampling and pretreatment

Granular sludge were sampled from the top, middle and/or bottom part of three EGSB reactors on different days (Table S1). All samples were washed with PBS and then centrifuged at 10000×g for 3 minutes. The supernatant was removed in order to reduce the bioavailability during biomass storage. All samples were stored at -25 ^{o}C until DNA extraction.

DNA extraction and 454-pyrosequencing

16S rDNA of biomass samples were extracted using a MoBio UltraClean Miocrobial DNA isolation kit (MoBIO Laboratories, Inc., CA, USA) following the protocol. In brief, a combination of heat, detergent, and mechanical force against specialized beads was involved in this process. DNA isolation was confirmed by agarose gel electrophoresis and the concentration of DNA was measured by using a Nanodrop 1000 equipment (Thermo Scientific, Waltham, MA, USA). The pyrosequencing of 16S rDNA gene was performed at Research and Testing Laboratory (Lubbock, TX, USA) using a Roche 454 GS-FLX facility (454 Life Science, Branford, CT, USA) with titanium chemistry. Universal primers U515F (GTG CCA GCM GCC GCG GTA A) and U1071R (GAR CTG RCG RCR RCC ATG CA) [1] were used in pyrosequencing. Post-processing and analysis of pyrosequencing results,

including chimera removal and taxonomic classification were performed by a Quantitative Insights into Microbial Ecology (QIIME) pipeline, version 1.7.0 [2].

Real-time quantitative polymerase chain reaction (qPCR)

Real-time qPCR was conducted using 16S rDNA gene primer pairs for both bacteria and archaea (see Table S2). A typical reaction system had a total volume of 20 µL, containing 10 µL SGExcel FastSYBR Mixture (with ROX) (Sangon Biotech, Shanghai, China), 0.2µL of each primer, 8.6µL of ddH_2O and 1 µL of DNA template. Amplification was applied by an ABI 7500 instrument (Foster City, CA) under corresponding programs for bacteria or archaea (Table S2). Standard curves were generated by tenfold diluting the standard plasmids to obtain a series of concentrations ranging from 10^4 to 10^{10} copies of plasmid DNA. The standard plasmids were constructed by cloning fragments obtained from PCR amplification of genomic DNA from total DNA (for both bacteria and archaea) from all EGSB reactors. Melt curve analysis was performed, confirming that primer dimers did not interfere with signal detection and primer binding was specific. Each sample was run in triplicate wells on 96-well plates.

Reference

1. Y. Wang and P.-Y. Qian, *PloS one*, 2009, 4, e7401.
2. J. G. Caporaso, J. Kuczynski, J. Stombaugh, K. Bittinger, F. D. Bushman, E. K. Costello, N. Fierer, A. G. Pena, J. K. Goodrich, J. I. Gordon, G. A. Huttley, S. T. Kelley, D. Knights, J. E. Koenig, R. E. Ley, C. A. Lozupone, D. McDonald, B. D. Muegge, M. Pirrung, J. Reeder, J. R. Sevinsky, P. J. Turnbaugh, W. A. Walters, J. Widmann, T. Yatsunenko, J. Zaneveld and R. Knight, *Nature methods*, 2010, 7, 335-336.

Supplementary Tables

Table S1 Real quantity and relative abundance of hydrogenotrophic and aceticlastic methanogens

Date	Hydrogenotrophic methanogen		Aceticlastic methanogens	
	Quantity (×10^9 gene copies per gram VS)	Relative abundance (%)	Quantity (×10^9 gene copies per gram VS)	Relative abundance (%)
One-stage BSG-EGSB				
Inoculum	73	5.5	41	3.1
Day 74	1	0.8	23	13.1
Two-stage BSG-EGSB				
Inoculum	73	5.5	41	3.1
Day 8	107	2.2	7	0.2
Day 113	725	6.3	360	3.1
PM-EGSB				
Inoculum	73	5.5	41	3.1
Day 47	95	1.6	20	0.3
Day 108	143	1.2	6	0.1
Day 173	203	3.6	4	0.1
Day 245	640	1.7	N.D.	N.D.

N.D. means the values are lower than the detection limit and thus are not detectable.

Table S2 Primers and heating programs for qPCR

Target kingdom	Name	Sequence (5'-3')	
Bacteria	BAC516F	TGCCAGCAGCCGCGGTAATAC	
	BAC805R	GACTACCAGGGTATCTAATCC	
Archaea	ARC787F	ATTAGATACCCSBGTAGTCC	
	ARC1059R	GCCATGCACCWCCTCT	
qPCR program			
Bacteria	1 min at 95 ^{o}C	40 cycles of 15s at 95°C, 30s at 53°C, 45s at 72°C	Melting curve analysis: denaturation of 1 min at 95 ^{o}C; cooling of 1 min at 55 ^{o}C; then 95°C for 15s and 60°C for 15s
Archaea		40 cycles of 10s at 95°C, 30s at 61°C, 45s at 72°C	
